# Evaluation of Machine Learning Techniques to Predict the Likelihood of Mental Health Conditions Following a First mTBI

**DOI:** 10.3389/fneur.2021.769819

**Published:** 2022-02-02

**Authors:** Filip Dabek, Peter Hoover, Kendra Jorgensen-Wagers, Tim Wu, Jesus J. Caban

**Affiliations:** ^1^National Intrepid Center of Excellence (NICoE), Bethesda, MD, United States; ^2^Computer Science Department, University of Maryland, Baltimore, MD, United States; ^3^Landstuhl Regional Medical Center, Landstuhl, Germany

**Keywords:** mild traumatic brain injury (mTBI), mental health, machine learning, data science, predictive modeling, forecasting

## Abstract

**Objective:**

Limited research has evaluated the utility of machine learning models and longitudinal data from electronic health records (EHR) to forecast mental health outcomes following a traumatic brain injury (TBI). The objective of this study is to assess various data science and machine learning techniques and determine their efficacy in forecasting mental health (MH) conditions among active duty Service Members (SMs) following a first diagnosis of mild traumatic brain injury (mTBI).

**Materials and Methods:**

Patient demographics and encounter metadata of 35,451 active duty SMs who have sustained an initial mTBI, as documented within the EHR, were obtained. All encounter records from a year prior and post index mTBI date were collected. Patient demographics, ICD-9-CM and ICD-10 codes, enhanced diagnostic related groups, and other risk factors estimated from the year prior to index mTBI were utilized to develop a feature vector representative of each patient. To embed temporal information into the feature vector, various window configurations were devised. Finally, the presence or absence of mental health conditions post mTBI index date were used as the outcomes variable for the models.

**Results:**

When evaluating the machine learning models, neural network techniques showed the best overall performance in identifying patients with new or persistent mental health conditions post mTBI. Various window configurations were tested and results show that dividing the observation window into three distinct date windows [−365:−30, −30:0, 0:14] provided the best performance. Overall, the models described in this paper identified the likelihood of developing MH conditions at [14:90] days post-mTBI with an accuracy of 88.2%, an AUC of 0.82, and AUC-PR of 0.66.

**Discussion:**

Through the development and evaluation of different machine learning models we have validated the feasibility of designing algorithms to forecast the likelihood of developing mental health conditions after the first mTBI. Patient attributes including demographics, symptomatology, and other known risk factors proved to be effective features to employ when training ML models for mTBI patients. When patient attributes and features are estimated at different time window, the overall performance increase illustrating the importance of embedding temporal information into the models. The addition of temporal information not only improved model performance, but also increased interpretability and clinical utility.

**Conclusion:**

Predictive analytics can be a valuable tool for understanding the effects of mTBI, particularly when identifying those individuals at risk of negative outcomes. The translation of these models from retrospective study into real-world validation models is imperative in the mitigation of negative outcomes with appropriate and timely interventions.

## 1. Introduction

Previous estimates suggest that 15–22% of all Service Members (SMs) have sustained a mild traumatic brain injury (mTBI) (1, 2). Many SMs develop persistent symptoms such as headaches, sleep disturbances, cognitive deficits, as well as changes in mood and behavior (3–5). These symptoms can often be further compounded due to the environment in which these injuries were sustained, particularly among combat-related mTBIs in which co-morbid conditions such as posttraumatic stress disorder (PTSD) and other mental health (MH) conditions are prevalent (6–8). As such, SMs and Veterans who have sustained an mTBI are at greater risk for developing MH conditions (8).

Despite best practices for treating SMs and Veterans with mTBI (9), prognosticating recovery from mTBI remains challenging. Multiple factors can affect symptom progression, including characteristics of the injury itself (10–12), as well as premorbid health conditions (10, 13, 14). Research has shown that the presence of co-morbid conditions, particularly mental health conditions, can often delay and further complicate the recovery of mTBI (15, 16). As such, the ability to identify individuals at risk for developing certain symptoms and anticipating their needs can greatly improve an individuals' outcome. Having the appropriate and timely interventions can assist in a faster resolution of symptoms and a quicker recovery (13).

In the field of data science for healthcare, the development of predictive models focuses on creating algorithms and devising methods that can be used to predict the likelihood of occurrence or recurrence of a particular event, such as a specific clinical diagnosis or other negative outcomes. During the last decade there has been a significant increase in popularity of developing tools for predicting outcomes at the level of the individual patient (17). Unfortunately, most models rely on regression techniques which result in a regression formula that often simplifies the forecast into a general risk score factor. Regression techniques often are sensitive to outliers and the data must be independent. Given those limitations and the continued growth of standardized clinical data for mTBI patients, additional research is needed to assess the applicability of advanced machine learning (ML) techniques into predicting the probability of obtaining a mental health diagnosis.

This paper evaluates various methods and techniques in an attempt to anticipate the outcomes of mTBI. By leveraging machine learning models and longitudinal EHR data, we hope to assist clinicians in identifying those individuals at risk for negative outcomes.

## 2. Background and Significance

Healthcare providers have long relied on data to understand the conditions and prognosis of a patient. The increased availability of health-related digital data has allowed for the exploration and evaluation of clinically-relevant issues on a much broader scale. Researchers and clinicians are no longer limited by finite sample sizes, often a constraint in many traditional cohort studies. The ability to analyze larger sample populations enables for more generalized results, due to diverse populations and settings, along with improved statistical power (18, 19). This has important implications when devising policies and establishing evidence-based clinical practices (20).

Through the widespread adoption of EHR systems, the rapid growth of healthcare data, and the steps taken by many hospital organizations to integrate different analytical tools within their clinical workflow; providers and administrators can now have greater insight into the etiologies of disease and subsequent outcomes (21). Currently, many analytical tools that have been integrated into the clinical workflow and are used to forecast specific clinical events such as hospital readmission, cardiovascular conditions, cost of the patient, faud, and negative outcomes within well-defined conditions (22, 23).

Within TBI research, the development of such forecasting tools have furthered our understanding of the various clinical presentations of concussive events and have helped with the evaluation of treatment efficacy (24). However, much existing research has focused on estimating the importance of *risk factors* and their association with particular outcomes (25–27).

Through the utilization of longitudinal EHR data and the advancement of data science, we now have the ability to expand upon previous techniques and incorporate a richer set of clinical characteristics. Recently, there has been an increased popularity in the development of these clinical predictive models to assist clinicians in anticipating outcomes for an individual patient (28, 29). Data science enables the use of structured and unstructured EHR data to develop a large set of features that models can use to perform classifications tasks (17). This increased flexibility is particularly important when considering the temporal aspect of events given that the time and sequence of events can play a vital role when evaluating patient outcomes. Recent research has stressed the importance of temporal events in predictive analytics (30, 31). Furthermore, data science techniques such as feature selection techniques can estimate and even select particular variables based upon predictive power. Dependent upon the specific task and its intention, the relevancy of features can greatly vary, and we can rely upon machine learning and data science techniques to assist in this evaluation.

As the quantity and complexity of the healthcare data collected for patients continues to increase, healthcare providers must embrace modern techniques that can assist with understanding a patients' condition and help evaluate the likelihood of various outcomes. This paper evaluates the utility of machine learning models and longitudinal EHR data as a mechanism to assist clinicians in identifying individuals at risk for developing mental health conditions. Patient demographics, encounter metadata, and data science algorithms are used to create clinical prediction models that can provide insight into potential needs of the patients. Throughout the paper, various supervised machine learning models were employed including logistic regression, support vector machines (SVMs), and neural network (NN) to develop predictive models capable of identifying patients at increased risk. In addition, multi-dimensional feature vectors and optimized observation windows were used to assist providers at identifying patients at different time intervals. This work serves as the foundation of some prospective work planned to validate the use of data science in TBI.

## 3. Objective

The objective of this study was to evaluate the utility of machine learning models and longitudinal EHR data to predict the likelihood of developing mental health (MH) conditions following the first diagnosis of mTBI. Electronic health record metadata, healthcare utilization, and preexisting conditions were utilized to generate a unique description of every patient, train different models, and perform evaluation of different approaches to determine the likelihood of developing mental health condition 1 year following injury.

## 4. Materials and Methods

### 4.1. Data Source

Direct care records were accessed through the Comprehensive Ambulatory Provider Encounter Record (CAPER) data file within the Military Health System Data Repository (MDR). For each encounter, metadata was extracted, which included International Classification of Diseases (ICD-9 & 10) diagnostic codes, provider types, procedural terminology codes, clinic codes, and demographics.

As a retrospective study, a waiver of documentation of informed consent was requested and approved for this study by the Walter Reed Institutional Review Board under IRB protocol #374953. All identifiable data was removed prior to analysis.

### 4.2. Sample Population

Our sample population included active duty SMs who had sustained an mTBI, as diagnosed by a healthcare provider adhering to VA/DoD criteria (9). In order to qualify for the study, each patient's direct care encounters had to be documented in the military EHR between 2005 and 2018. The earliest date of mTBI diagnosis for each patient was defined as the index date.

To guarantee a complete longitudinal dataset, only SMs with encounter data greater than 1 year prior to and 1 year post-injury were included. To ensure that the date of diagnosis accurately reflects the date of injury, patients were excluded whose initial mTBI was defined as a *personal history* of TBI (e.g., ICD-10-CM code Z87.820, ICD-9-CM code V15.52), a diagnostic category used to reflect a previous TBI regardless of when it occurred. Furthermore, to establish that results were not confounded by subsequent injuries, patients were excluded who had a more severe diagnosis of TBI up to 1 year after initial injury date.

**Figure 1:**
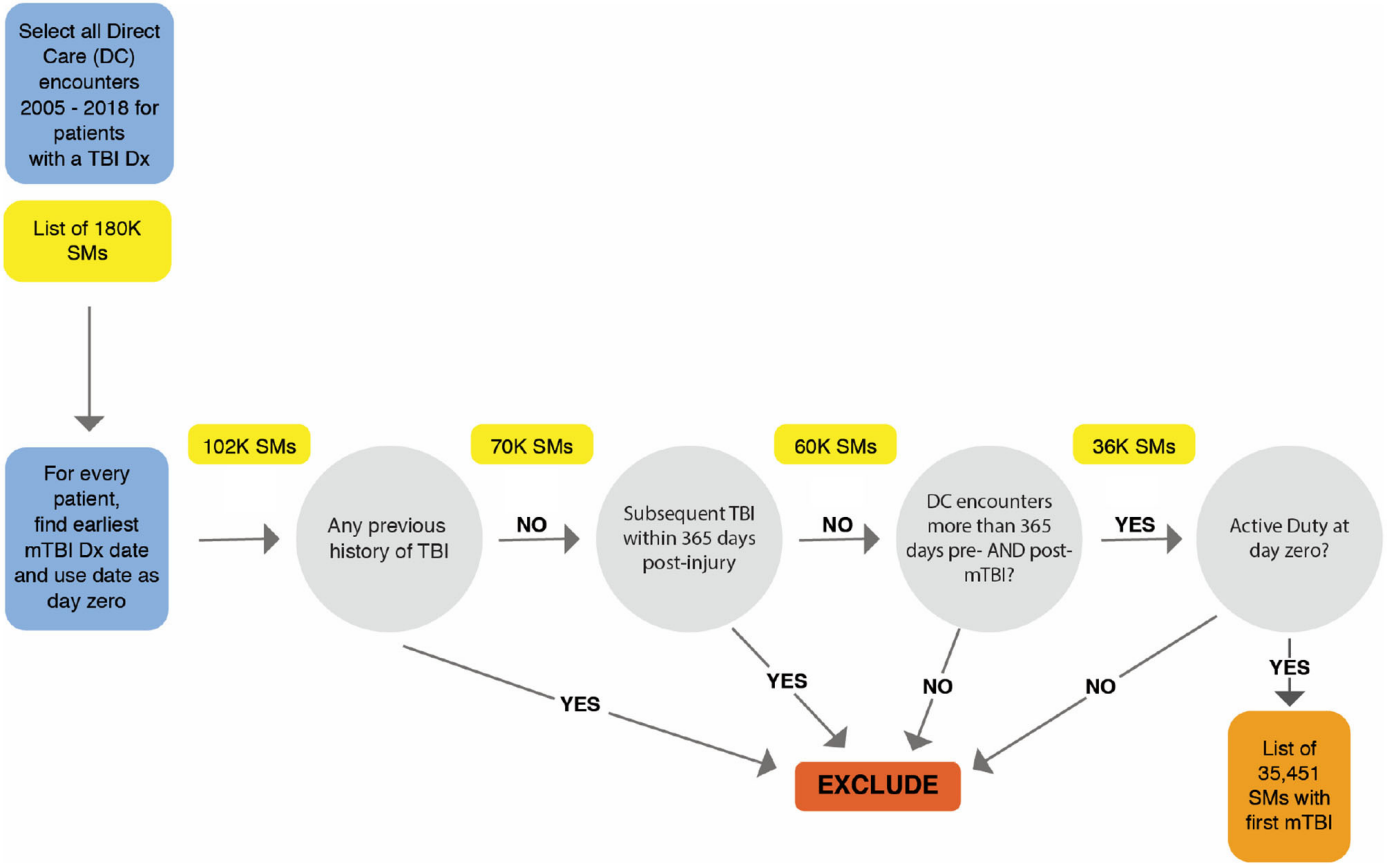
Methodology for selecting sample population.

The dataset consisted of 35,451 active duty SMs whose first mTBI was documented by a healthcare provider within the MHS, initially amounting to 4,901,840 direct care outpatient clinical encounters. Utilizing only encounters 365 days before and after mTBI diagnosis date, 1,369,740 encounter records were assessed. Figure 1 illustrates the process and inclusion criteria for determining the sample population.

### 4.3. Feature Vectors

In data science and machine learning, *features* are individual independent variables directly extracted or derived from the raw data that are used to described the unique characteristics of the object under consideration. A *feature vector* is the n-dimensional vector of independent features that is used to describe a given object.

One of the key components of designing ML models is using domain knowledge to extract features. For this particular effort, with our sample population defined, the attributes that were included within the final feature vectors can be categorized into three domains: *demographics, symptomatology*, and *other risk factors*. Those feature vectors were generated only from the data from the observation period.

#### 4.3.1. Demographics Features

Although various demographic attributes were available within the EHR, due to collinearity among variables, our final dataset included demographics specific to age, gender, and branch of service (BoS). Existing literature has supported the utility of BoS, as different branches experience, diagnose, and/or treat TBIs differently (32).

#### 4.3.2. Symptomatology

To indicate and quantify preexisting conditions, ICD-9-CM and ICD-10 codes associated with common mTBI symptoms and complaints (33–35), were obtained. These codes were then grouped into Enhanced Diagnostic Related Groups (EDRGs) based on related symptomatology. For example, the Anxiety EDRG included codes such as “300.02-Generalized Anxiety Disorder,” “300.01-Panic Disorder,” and “F41.9-Anxiety Disorder, unspecified.” To support the grouping and classification process, the Expanded Diagnostic Clusters (EDC) used in the John Hopkins Adjusted Clinical Groups (ACG) model (36), were employed. To evaluate symptom presence, EDRGs were then mapped to related encounter-level diagnostic codes and the frequency for each EDRG was then computed. Encounter records within the year prior to index mTBI were utilized to obtain these counts.

EDRGs related to mental health (e.g., anxiety, depression, adjustment disorder, etc.) were extracted from encounter records within the predictive timeframe to define the outcome variable of presence or not of MH conditions following mTBI. For a complete list of diagnosis codes, see Supplementary Table 1.

#### 4.3.3. Risk Factors Features

A critical component of feature engineering is the process of using domain knowledge to extract features. Previous literature has identified key risk factors for developing mental health conditions (37). As a result, diagnosis codes specific to suicide attempts/ideation and substance abuse disorders were obtained from the Clinical Classifications Software (CCS) mapping created by the Agency for Healthcare Research and Quality (AHRQ) (38, 39). Encounter records within the year prior to index mTBI were used to identify those with preexisting risk factors. The frequency of these risk factors were then quantified and included as part of the feature vector.

Once the different sub-components of our feature vectors were generated, they were combined into an n-dimensional feature vector:


$$\begin{array}{l} P_{1}=\left\{D_{1},D_{2},D_{3},\ldots \right\}\cup \left\{S_{1},S_{2},S_{3},\ldots \right\}\cup \left\{R_{1},R_{2},R_{3},\ldots \right\} \end{array}$$

where *P*_1_ is a sample patient, *D*_*i*_ the features associated with demographics, *S*_*i*_ features associated with symptomatology, and *R*_*i*_ features associated with risk factors. For a complete list of the different codes that were used for the different EDRGs, see Supplementary Table 2.

### 4.4. Window Configurations

As discussed, we recognize the temporal significance of preexisting symptoms and the specific sequence these conditions happen. Conditions present 1 year pre-mTBI might hold different clinical relevance than a condition present at time of injury. For a model to better learn of these temporal associations and identify their significance, we sought to build and devise various observation window configurations.

**Figure 2:**
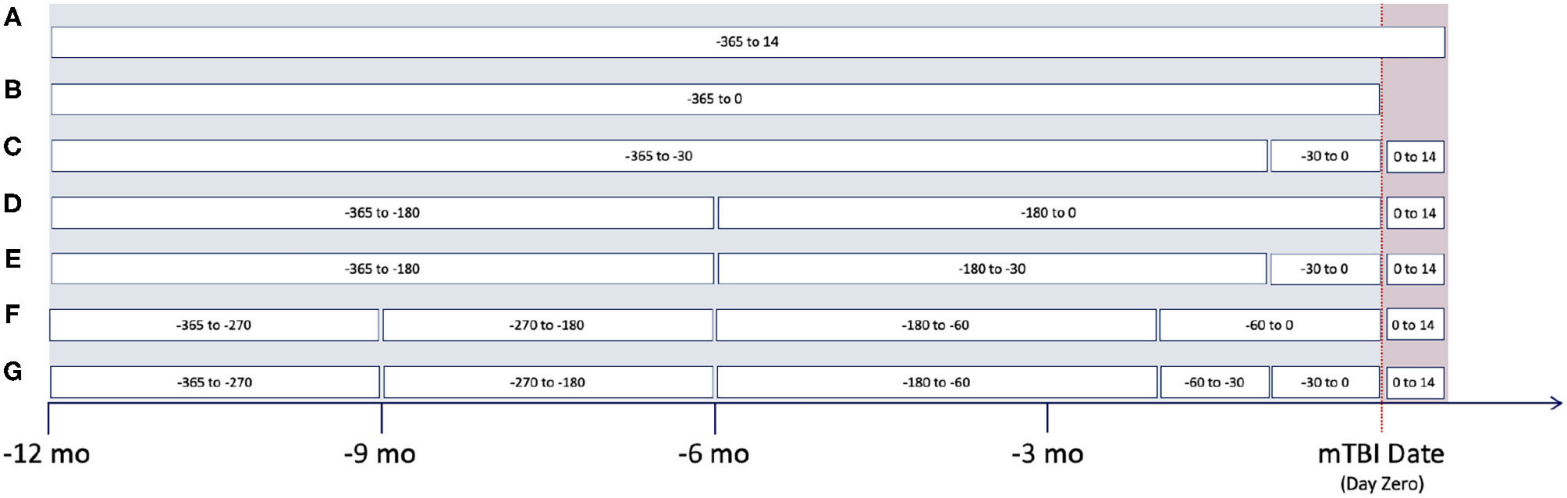
Different window configurations for the observation period.

Based upon clinical knowledge, we first divided the entire observation period into distinct intervals, referring to them as “window configurations.” In the past, we had found that 1-month (30-day) intervals were most effective for splitting EHR data (40). However, as historical EHR data can be relatively sparse, various other window configurations were created. These adaptive configurations grouped observation windows with sparse clinical data, typically those time periods at greatest distance from mTBI date. Figure 2 provides an illustration of the various window configurations, A through G, that we employed for this particular project.

**Figure 3:**
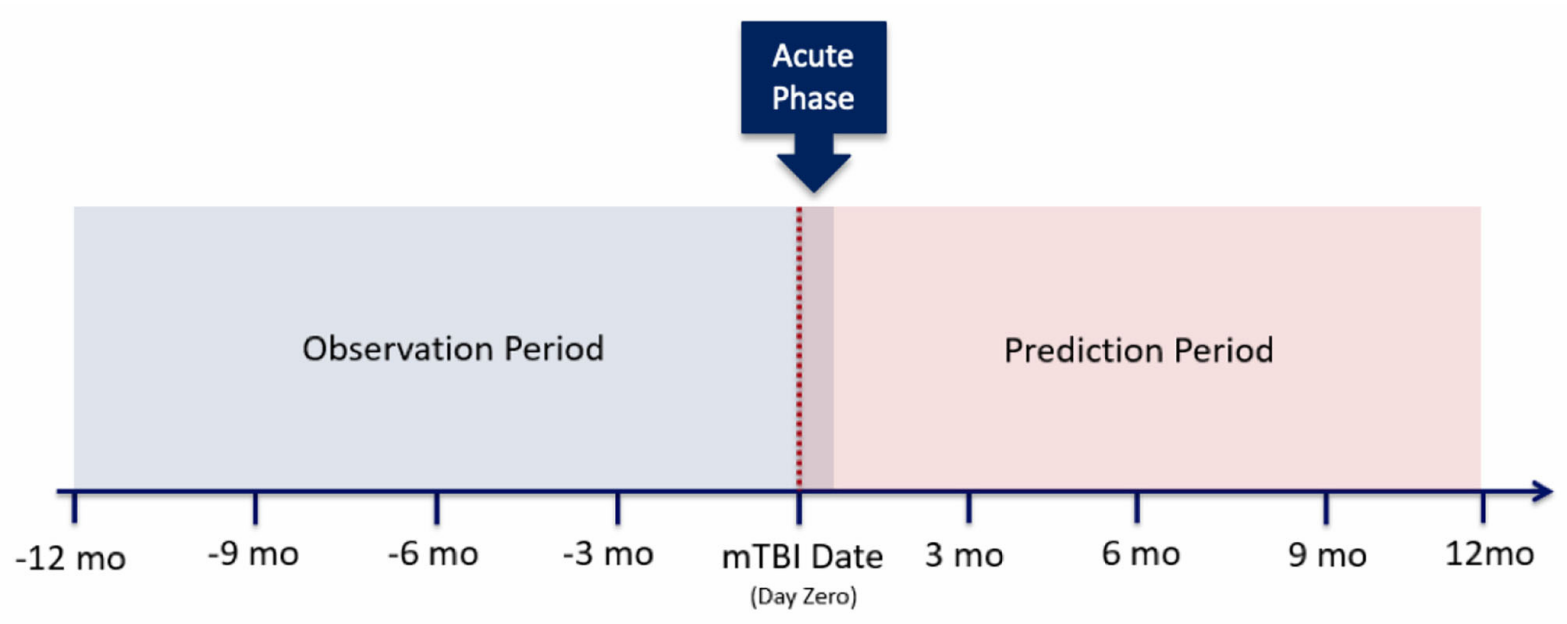
Patient's clinical trajectory, split into observation and prediction periods.

As noted within Figure 2, several of these configurations utilize the first 14 days post-mTBI, often referred to as the “acute phase.” We chose to include this window as the first 14 days post-TBI are considered the crucial period of treatment for understanding the recovery and long term trajectory of a patient (41). Therefore, our models leveraged data between [−365:14] days, with respect to mTBI date, for the prediction of events between [14:365] (Figure 3).

It's important to note that by adding different configuration windows, the feature vector now is estimated for each window, thus resulting in vectors like


$$\begin{array}{l} P_{1}=\left\{D_{1},D_{2},D_{3},\ldots \right\}\cup \left\{S_{1}^{w1},S_{2}^{w1},S_{3}^{w1},\ldots \right\}\\ \;\cup \left\{S_{1}^{w2},S_{2}^{w2},S_{3}^{w2},\ldots \right\}\cup \ldots \\ \;\{R_{1}^{w1},R_{2}^{w1},R_{3}^{w1},\ldots \}\cup \left\{R_{1}^{w2},R_{2}^{w2},R_{3}^{w2},\ldots \right\} \end{array}$$

where *P*_1_ is a sample patient, *D*_*i*_ the features associated with demographics, $S_{i}^{w1}$ features associated with symptomatology for window 1, and $R_{i}^{w2}$ features associated with risk factors for window 2.

As detailed above, each patient within the dataset had attributes specific to three main categories (demographics, risk factors, and symptoms). Demographics included three distinct attributes: age, gender, and branch of service. Risk factors included two attributes: substance abuse and suicide attempts/ideation. Lastly, symptoms included 14 attributes, each representing a distinct symptom. As such, each patient had 19 attributes by default. The number of attributes may increase depending upon the number of window configurations used within the model, as symptoms and risk factors were calculated with respect to the observation windows. For example, if three observation windows were used (Window Configuration C), patients would then have 51 attributes; 14 symptoms and two risk factors for each individual window.

### 4.5. Models and Metrics

For this study, various supervised machine learning models were employed. Supervised algorithms are those that learn from “labeled” data and once trained are tested and used to predict the classification of “unlabeled” data. As each model type has its own benefits and limitations, it is important to evaluate and compare different models and their performances. For completeness, logistic regression, support vector machines (SVM), random forests, elastic nets, adaptive boosting (AdaBoost), and neural networks were all employed. To compensate for class imbalance, Synthetic Minority Over-sampling Technique (SMOTE) (42), was also employed on the training set. These techniques were used in combination to the different model types.

To test and perform a thorough evaluation of the different models, we split the original dataset into two groups using a 80:20 split: *training* and *testing*. The *training* set was then split (training/validation) to be used as input variables to create the model and estimate parameters. The *testing* dataset was used to validate, compare, and optimize the different models.

To assist in the tuning of hyper-parameters among the varying models, grid search was employed on the training data for the non-neural network approaches. For the neural networks, the Adam optimizer was used along with its default learning rate. The network consisted of an input with its dimension in respect to the size of the corresponding window size, a hidden layer of size 19 with a tanh activation function, and then a single output node that used a sigmoid activation function. The network was trained using the binary cross entropy loss.

The receiver operating characteristic (ROC) curve, the area under curve (AUC), and the area under the precision-recall curve (AUC-PR) were utilized for comparison and evaluation. The ROC curve is created by plotting the true positive rate (i.e., probability that an actual positive case will test positive) against the false positive rate (i.e., when the truth is negative, but the model predicts a positive) at various threshold settings. The precision-recall curve summarizes the trade-off between optimizing for precision (i.e., positive predictive value) against recall (i.e., sensitivity). As it is difficult for a model to perform well under both metrics, the precision and recall values are often inversely proportionate.

The AUC-PR metric summarizes this tradeoff into a singular value for easy model comparison. Of note, the AUC-PR metric is a different measurement than the AUC, has been found to be more informative in the context of imbalanced binary classification tasks (43, 44), and will typically have smaller values ranging from about 0.2 to 0.5 (45, 46).

The utilization of ROC, AUC, and AUC-PR is important to validate models with class imbalance limitations—when the dataset has an unequal distribution of classes in the training dataset. Class imbalance is common in healthcare given that often there will be a majority of cases that are negative and a minority group that are positive for a particular condition.

For the development of feature vectors and the construction of models, Python 3.6.6 was used (47). Accompanying packages including Pandas 0.24.4, Numpy 1.15, and Imbalanced-learn 0.4.3 were used for the manipulation, cleaning, and resampling of data (48–50). Scikit-learn 0.20.1, Keras 2.2.4, SciPy 1.1.0, and Matplotlib 3.4.1 were utilized to create models and evaluate performance (51–54).

## 5. Results

### 5.1. Demographics

For this analysis, a total of 35,451 SMs met the inclusion/exclusion criteria (Table 1). This resulted in a sample population of 29,736 men (83.9%), with the majority between the ages of 17 and 24 (47.6%). Examining military characteristics, over half of the sample population was from the United States Army (55.4%), while 50.7% were classified as Junior Enlisted. Chi-square was performed to validate that sample population is representative of the active duty TBI population.

**Table 1:** Summary of patient demographics and prevalence of preexisting conditions (*n* = 35,451).

		***n***	**%**
Gender	Male	29,736	83.9
	Female	5,715	16.1
Age	17–24	16,890	47.6
	25–34	12,816	36.2
	35–44	4,665	13.2
	45+	1,080	3.0
Service branch	Army	19,778	55.8
	Air force	6,204	17.5
	Marine corps	4,396	12.4
	Navy	4,491	12.7
	Other	582	1.6
Rank	Cadet	1,148	3.2
	Enlisted, Junior	17,988	50.7
	Enlisted, Senior	12,401	35
	Officer, Junior	2,131	6.0
	Officer, Senior	1,021	2.9
	Officer, Warrant	322	0.9
	Unknown	440	1.2
Preexisting conditions	Anxiety	2,551	7.2
	Appetite	3,005	8.5
	Audiology	1,745	4.9
	Balance/Dizziness	1,289	3.6
	Cognitive	1,972	5.6
	Depression	2,401	6.8
	Fatigue	828	2.3
	Headaches	7,871	22.2
	Musculoskeletal	21,531	60.7
	Neurology	3,338	9.4
	Psychology, Other	7,201	20.3
	PTSD	1,955	5.5
	Sleep	3,718	10.5
	Substance abuse	2,008	5.6
	Suicide ideation/Attempt	324	0.9
	Vision	1,160	3.3

In the evaluation of preexisting conditions, musculoskeletal were most prominent among this population, (60.7%), followed by headaches (22.2%). Examining the MH-related conditions, 6.8% of the population were diagnosed with depression with 20.3% diagnosed with a Psychological, Other condition. Regarding the outcome variable, 32.1% of the sample population were diagnosed with at least one MH condition within the year following the index mTBI (Table 2).

**Table 2:** Proportion of service members with mental health conditions.

	**Remitting**	**New onset**	**Persistent**	**Not diagnosed**
Anxiety	1,105 (3.1%)	2,141 (6%)	1,446 (4.1%)	30,759 (86.8%)
Depression	1,117 (3.2%)	1,959 (5.5%)	1,284 (3.6%)	31,091 (87.7%)
Psychology, Other	3,256 (9.2%)	4,786 (13.5%)	3,945 (11.1%)	23,464 (66.2%)
PTSD	548 (1.5%)	1,536 (4.3%)	1,407 (4%)	31,960 (90.2%)
Substance abuse	1,037 (2.9%)	1,660 (4.7%)	971 (2.7%)	31,783 (89.7%)
Suicide ideation/Attempt	286 (0.8%)	401 (1.1%)	38 (0.1%)	34,726 (98.0%)

*Remitting, conditions present only prior to mTBI; New Onset, newly diagnosed conditions; Persistent, conditions present before and after mTBI*.

### 5.2. Feature Importance Ranking

In machine learning, feature importance ranking is the process that measures the contribution of individual features into the model. As part of the initial model development stages, we first evaluated the impact of embedding different feature vectors. We trained models to predict the year post-mTBI [14:365] and incrementally added features (e.g., demographics, symptoms, and risk factors). Table 3 contains the results for models (SVM, neural network), with the addition of the logistic regression as a baseline comparison. Assessing the logistic regression model, for example, utilizing only patient demographics resulted in an accuracy of 68.0%, an AUC of 0.51, and an AUC-PR of 0.33. By adding risk factors as input features, we notice an improvement in model performance; as noted by small increase in accuracy, AUC, and AUC-PR. However, when adding the patient symptomatology to the models, performance significantly improved to 74.9%. Through the iterative addition of features, the incremental predictive power of each could be seen through the boosts in performance metrics across these model types.

**Table 3:** Feature importance derived from model performance with iterative addition of features where D are demographics, R are risk factors, and S are symptoms.

	**Logistic regression**			**SVM**			**Neural network**		
**Feature**	**ACC**	**AUC**	**AUC-PR**	**ACC**	**AUC**	**AUC-PR**	**ACC**	**AUC**	**AUC-PR**
D	68.0	0.51	0.33	67.7	0.5	0.32	68.0	0.61	0.40
D + R	69.6	0.53	0.36	69.8	0.54	0.36	69.8	0.65	0.49
D + R + S	74.9	0.62	0.46	76.8	0.68	0.50	77.0	0.75	0.65

*ACC, Accuracy; AUC, Area Under Curve; AUC-PR, Area Under Curve Precision Recall*.

### 5.3. Model Types and Variations

With the inclusion of all feature vectors, we evaluated the performance of the different model types and variations previously outlined. The model types varied and we found the best performing for comparison to be logistic regression, SVM, and neural network models. Table 3 details the original results utilizing the full feature vectors (demographics, risk factors, symptoms) against these models. With these models, we attempted to employ SMOTE to assist with the class imbalance. However, improvement in model performances were not observed.

First, we can see that after using all the features logistic regression performs with a 74.9% accuracy and 0.46 AUC-PR. By leveraging more advanced techniques such as SVMs, we can see an improvement to 76.8% accuracy and the 0.50 AUC-PR. Finally, when we apply state-of-the-art techniques like neural networks we can see an improvement to 77% accuracy and 0.65 AUC-PR.

With regards to computational costs, the logistic regression and SVM were consistently trained within 13–14 s, irrespective of the number of observation windows. AdaBoost and the random forest trained within 1 s while the neural network took approximately 23 s to train a single observational window (window configuration A). When training the maximum number of windows, the neural network took 27.4 s.

### 5.4. Observation Window Configurations

To evaluate the effectiveness of window configurations and its impact on performance, we ran each configuration from Figure 2 against the various model types. The results from the logistic regression, SVM, and neural network are provided within Table 4. When comparing configuration A [−365:14] and B [−365:0], we see the importance of including the acute phase, 14 days post-mTBI, within our models; as noted by the differences in accuracies, AUC, and AUC-PR values. This underscores the impact of the acute phase on the trajectory of a patient as well as on the ability to accurately predict long-term patient outcomes. Evaluating Table 4, configuration C [−360:−30, −30:0, 0:14], provided the best performance. The accuracy, AUC, and AUC-PR substantially increased compared to configurations A and B. It appears that isolating those 30 days prior to mTBI seemed to provide the models with more significant information.

**Table 4:** Model performance on different window configurations for the observation period.

	**Logistic regression**			**SVM**			**Neural network**		
**Configuration**	**ACC**	**AUC**	**AUC-PR**	**ACC**	**AUC**	**AUC-PR**	**ACC**	**AUC**	**AUC-PR**
A	74.9	0.62	0.46	76.8	0.68	0.50	76.7	0.75	0.65
B	73.8	0.60	0.44	75.7	0.66	0.48	75.3	0.73	0.62
C	77.4	0.67	0.52	78.5	0.71	0.54	78.2	0.78	0.70
D	76.8	0.65	0.50	77.4	0.68	0.51	77.4	0.76	0.67
E	77.0	0.66	0.50	77.2	0.68	0.50	77.4	0.76	0.67
F	74.8	0.62	0.46	75.7	0.65	0.48	76.1	0.74	0.64
G	75.0	0.62	0.46	75.6	0.65	0.47	75.9	0.74	0.64

*ACC, Accuracy; AUC, Area Under Curve; AUC-PR, Area Under Curve Precision Recall*.

**Figure 4:**
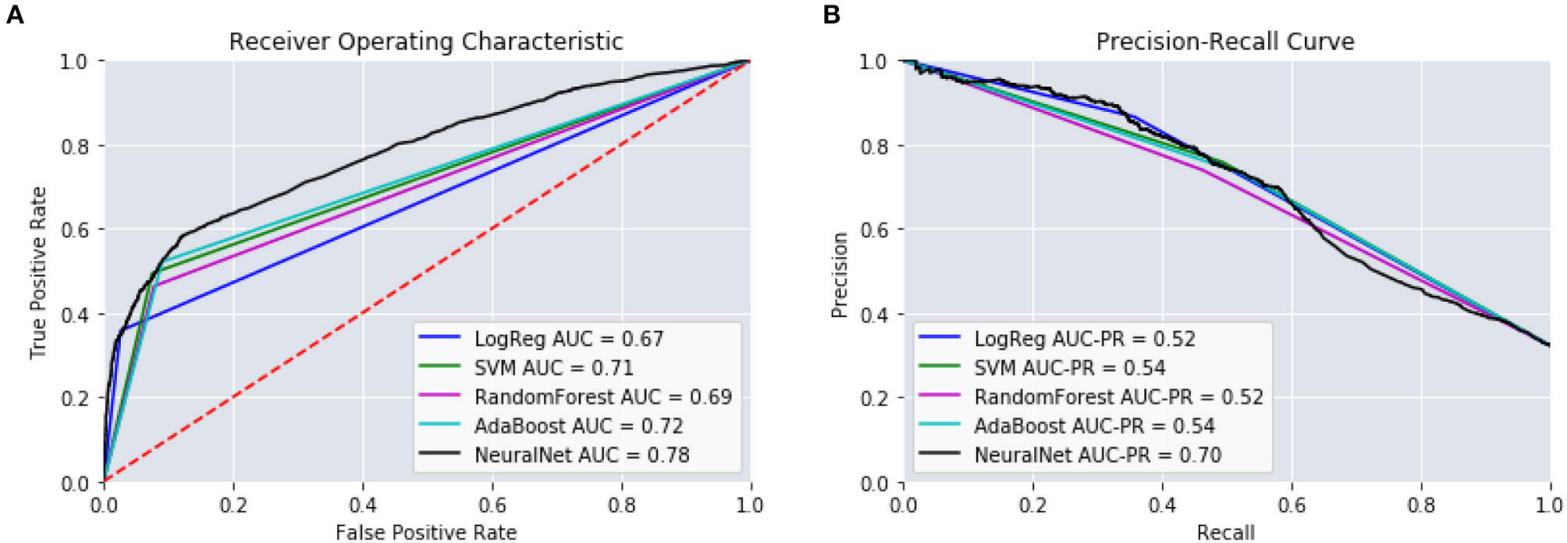
Different model types and their performance using window configuration C on predicting 14–365 days. **(A)** ROC Curves. **(B)** Precision-Recall Curves.

Dividing the observation period further, configurations D and E, the results of the models began to degrade. We can see that as the number of windows increases, configurations F and G, the performance of our models continues to drop. The sparseness of the data might have had an impact on reductions in model performance. Therefore, configuration C was chosen as the optimal window configuration among our model types. Figure 4 includes the AUC and AUC-PR curves for this configuration against all the different model types. It becomes apparent that many of these model types performed similarly, with the exception of the neural network which outperformed the other model types substantially.

### 5.5. Prediction Timeframes

**Figure 5:**
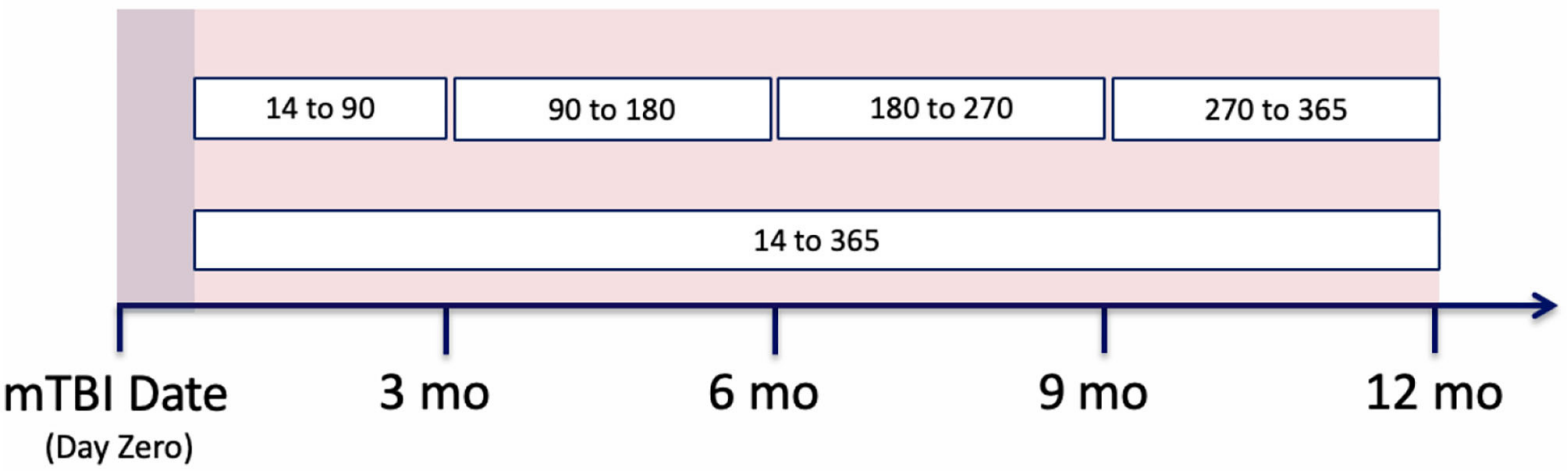
Prediction period split into different patients' clinical trajectories, split into observation and prediction periods. Then the observation period is further split into smaller window configurations.

While we were able to develop models that predict the likelihood of mental health symptoms within the year post-mTBI, we recognize the utility of more finite prediction windows; predicting the likelihood of symptoms within smaller time frames. As such, our prediction period was divided into a set of smaller windows, including the first 3 months post-mTBI [14:90], the following 6 months [90:180], and the last 6 months [180:365] (Figure 5). Utilizing the various model types, along with the observation window configuration C, we predicted the likelihood of MH conditions within each interval.

**Figure 6:**
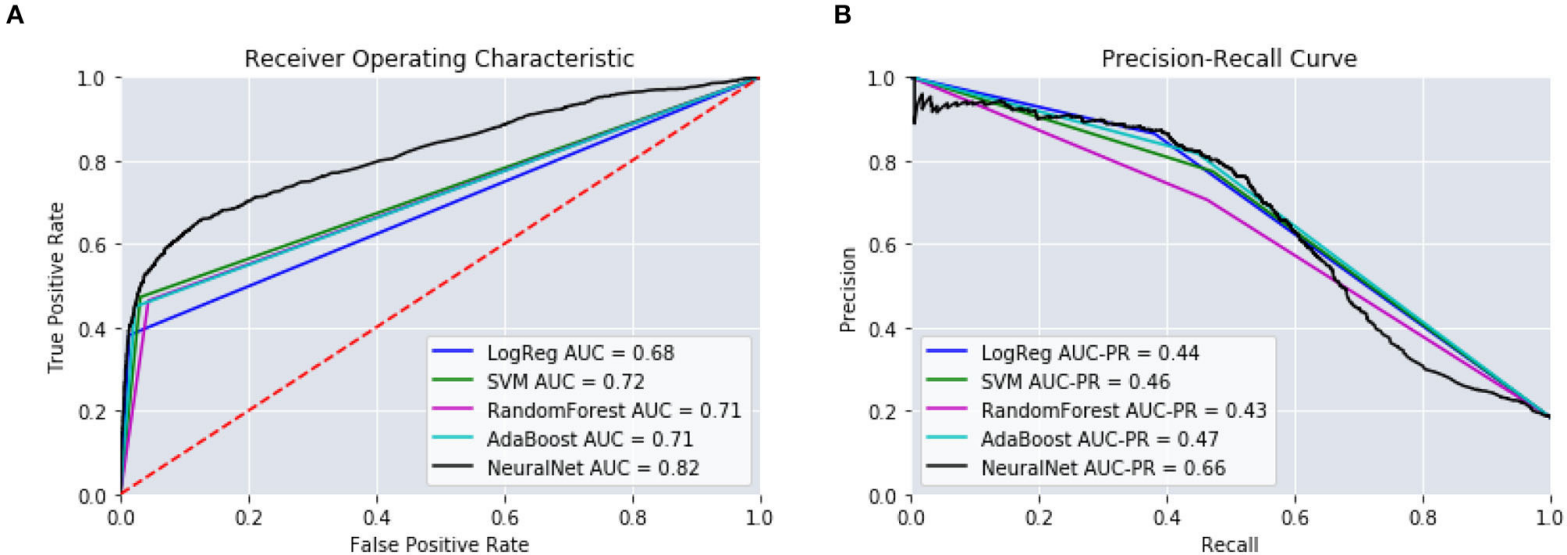
Performance of the predictive models using [14:90]. **(A)** ROC Curves. **(B)** Precision-Recall Curves.

Table 5 provides the results from these models. We can see that those intervals closest to the mTBI date, [14:90], obtained the best performances. The further from mTBI date, the lower the model accuracies. Figure 6 details the AUC and AUC-PR curves for predicting the onset of MH conditions within the [14:90] prediction window. Again, while many of the model types seemed to perform similarly, the neural network models performance stood out, with an accuracy of 88.2, an AUC of 0.82, and an AUC-PR of 0.66.

**Table 5:** Effect of splitting the prediction period into windows based on clinical insight.

	**Logistic regression**			**SVM**			**Neural network**		
**Window**	**ACC**	**AUC**	**AUC-PR**	**ACC**	**AUC**	**AUC-PR**	**ACC**	**AUC**	**AUC-PR**
14 to 365	85.32	0.59	0.28	85.6	0.62	0.31	86.1	0.78	0.53
14 to 90	87.5	0.68	0.44	87.7	0.72	0.46	88.2	0.82	0.66
90 to 180	89.0	0.56	0.19	89.1	0.58	0.21	89.3	0.78	0.44
180 to 270	83.8	0.56	0.24	84.2	0.60	0.28	84.6	0.75	0.46
270 to 365	80.9	0.54	0.25	81.5	0.58	0.28	80.8	0.72	0.46

*ACC, Accuracy; AUC, Area Under Curve; AUC-PR, Area Under Curve Precision Recall*.

## 6. Discussion

Through the development of feature vectors and the configuration of observation windows, this study assessed the capability of using EHR data to predict those at risk for negative mTBI outcomes. Due to the nature of the data, considerations were needed when devising our feature vectors. We first evaluated the impact of incrementally adding various variables. The inclusion of patient demographics, risk factors, and premorbid conditions increased model performance. In an attempt to provide temporal significance, we examined the benefit of dividing our observation period into distinct windows. After iterating through multiple configurations, dividing the observation period into three distinct windows provided the best performance.

With our best performing model, leveraging a neural network, we further attempted to predict the diagnosis of any mental health condition within distinct outcome windows. Encoding the observation windows into intervals allowed the models to assess and weigh the significance of each vector. Oftentimes those intervals closest to the mTBI date held greatest significance. This was particularly true when assessing those prediction intervals closest to the mTBI date, which exhibited the greatest accuracies.

Through these practices, we can better understand how EHR data can be leveraged to predict clinical outcomes. Though careful consideration is needed, the development of these tools and their deployment within clinical settings can provide great benefit to patients and clinicians alike. By understanding ones' risk of developing certain conditions or adverse outcomes, clinicians can provide prompt and timely interventions. This can aid in the mitigation of potential negative outcomes and the alteration of outcome trajectories. Furthermore, understanding which factors contribute to certain outcomes can help better understand the development and progression of disease.

With the utilization of EHR data, we can look forward to deploying and verifying our models within clinical settings. As this study leveraged a relatively clean dataset, further inclusion of feature vectors should be explored. Additional EHR data, including laboratory and radiology records could be considered. Furthermore, the inclusion of SMs with complex clinical histories is needed. Within the military population, it is not uncommon for SMs to experience repeated concussions, subsequent injuries, or other comorbidities. In order for these models to have clinical merit, they must be able to cater and adapt to various scopes and populations.

Furthermore, this paper detailed the retrospective application of these models. Future clinical application should be explored through prospective means. With the inclusion of additional features, applying our models prospectively will assist in further improving and validating our techniques. Clinicians could then verify such tools while enhancing clinical decision making.

## 7. Conclusion

Predictive analytics, specifically the use of neural networks, show promise in adopting data science models to identify the likelihood of developing mental health conditions following mTBI. These models can enable clinicians to not only identify at risk individuals, but to better anticipate patient needs and provide interventions to mitigate negative outcomes.

Translation of these models from retrospective constructs into real-world application is imperative. While this paper has demonstrated the technical feasibility of leveraging neural networks for TBI clinical application, on-going multi-site efforts are focusing on (i) optimizing the models to incorporate additional variables, (ii) implement interpretability to be able to incorporate the forecasting models into clinical ancillary applications, and (iii) use these retrospective data models into real-world prospective clinical environments.

## Data Availability Statement

The datasets presented in this article are not readily available because the Defense Health Agency (DHA) requires an approved Data Sharing Agreement (DSA) with potential users to confirm that data from the Military Health System (MHS) be used or disclosed in compliance with Department of Defense (DoD) privacy and security regulations. Requests to access the datasets should be directed to Jesus Caban, jesus.j.caban.civ@mail.mil.

## Ethics Statement

The studies involving human participants were reviewed and approved by Walter Reed National Military Medical Center. Written informed consent for participation was not required for this study in accordance with the national legislation and the institutional requirements.

## Author's Note

The views expressed in this article are those of the authors and do not necessarily reflect the official policy of the Department of Defense or the U.S. Government.

## Author Contributions

FD, PH, KJ-W, and JC developed methodology and outlined project. TW queried and extracted electronic healthcare record data. FD built and evaluated predictive models. FD and PH drafted manuscript. KJ-W and JC edited manuscript. All authors contributed to the article and approved the submitted version.

## Conflict of Interest

The authors declare that the research was conducted in the absence of any commercial or financial relationships that could be construed as a potential conflict of interest.

## Publisher's Note

All claims expressed in this article are solely those of the authors and do not necessarily represent those of their affiliated organizations, or those of the publisher, the editors and the reviewers. Any product that may be evaluated in this article, or claim that may be made by its manufacturer, is not guaranteed or endorsed by the publisher.
